# Frequency of *FGF14* intronic GAA repeat expansion in patients with multiple system atrophy and undiagnosed ataxia in the Japanese population

**DOI:** 10.1038/s41431-024-01743-3

**Published:** 2024-11-27

**Authors:** Toshiyuki Kakumoto, Kenta Orimo, Takashi Matsukawa, Jun Mitsui, Tomohiko Ishihara, Osamu Onodera, Yuta Suzuki, Shinichi Morishita, Japan Multiple System Atrophy Registry Consortium, Ayaka Chikada, Kenta Orimo, Takashi Matsukawa, Tatsushi Toda, Jun Mitsui, Hiroyufki Ishiura, Koji Abe, Toru Yamashita, Hidehiro Mizusawa, Yuji Takahashi, Masahisa Katsuno, Kazuhiro Hara, Osamu Onodera, Tomohiko Ishihara, Masayoshi Tada, Satoshi Kuwabara, Atsuhiko Sugiyama, Yoshitaka Yamanaka, Ryosuke Takahashi, Yusuke Sakato, Tomoyuki Ishimoto, Nobukatsu Sawamoto, Ritsuko Hanajima, Yasuhiro Watanabe, Hiroshi Takigawa, Tadashi Adachi, Hiroshi Takashima, Keiko Higashi, Junichi Kira, Ichiro Yabe, Masaaki Matsushima, Katsuhisa Ogata, Kinya Ishikawa, Yoichiro Nishida, Taro Ishiguro, Kokoro Ozaki, Tetsuya Nagata, Shoji Tsuji, Tatsushi Toda, Shoji Tsuji

**Affiliations:** 1 Department of Neurology, Graduate School of Medicine, The University of Tokyo, Tokyo, Japan; 2 Department of Precision Medicine Neurology, Graduate School of Medicine, The University of Tokyo, Tokyo, Japan; 3 Department of Neurology, Brain Research Institute, Niigata University, Niigata, Japan; 4 Advanced Treatment of Neurological Diseases Branch, Brain Research Institute, Niigata University, Niigata, Japan; 5 Department of Molecular Neuroscience, Brain Research Institute, Niigata University, Niigata, Japan; 6 Department of Computational Biology and Medical Sciences, Graduate School of Frontier Sciences, The University of Tokyo, Tokyo, Japan; 7 Institute of Medical Genomics, International University of Health and Welfare, Chiba, Japan; 8 Department of Neurology, Graduate School of Medicine, and Department of Precision Medicine Neurology, Graduate School of Medicine, The University of Tokyo, Tokyo, Japan; 9 Department of Neurology, Okayama University Graduate School of Medicine, Dentistry and Pharmaceutical Sciences, Okayama, Japan; 10 Department of Neurology, National Center Hospital, National Center of Neurology and Psychiatry, Tokyo, Japan; 11 Department of Neurology and Department of Clinical Research Education, Nagoya University Graduate School of Medicine, Nagoya, Japan; 12 Department of Neurology, Nagoya University Graduate School of Medicine, Nagoya, Japan; 13 Department of Neurology, Graduate School of Medicine, Chiba University, Chiba, Japan; 14 Department of Neurology, Kyoto University Graduate School of Medicine, Kyoto, Japan; 15 Department of Human Health Sciences, Kyoto University Graduate Schoolof Medicine, Kyoto, Japan; 16 Division of Neurology, Department of Brain and Neurosciences, Faculty of Medicine, Tottori University, Yonago, Japan; 17 Department of Neurology and Geriatrics, Graduate School of Medical and Dental Sciences, Kagoshima University, Kagoshima, Japan; 18 Department of Neurology, Graduate School of Medical Sciences, Kyushu University, Fukuoka, Japan; 19 Department of Neurology, Faculty of Medicine and Graduate School of Medicine, Hokkaido University, Hokkaido, Japan; 20 Department of Neurology, National Hospital Organization Higashisaitama Hospital, Saitama, Japan; 21 Department of Neurology and Neurological Science, Tokyo Medical and Dental University, Tokyo, Japan

**Keywords:** Clinical genetics, Spinocerebellar ataxia

## Abstract

Multiple system atrophy (MSA) is a neurodegenerative disorder characterized by autonomic nervous system dysfunction and cerebellar ataxia or parkinsonism. Recently, expanded GAA repeats (≥250 repeat units) in intron 1 of *FGF14* have been shown to be responsible for spinocerebellar ataxia type 27B (SCA27B), a late-onset ataxia with an autosomal dominant inheritance. Patients with SCA27B may also exhibit autonomic nervous system dysfunction, potentially overlapping with the clinical presentations of MSA patients. In this study, to explore the possible involvement of expanded GAA repeats in MSA, we investigated the frequencies of expanded GAA repeats in *FGF14* in 548 patients with MSA, 476 patients with undiagnosed ataxia, and 455 healthy individuals. To fully characterize the structures of the expanded GAA repeats, long-range PCR products suggesting the expansion of GAA repeats were further analyzed using a long-read sequencer. Of the 548 Japanese MSA patients, we identified one MSA patient (0.2%) carrying an expanded repeat with (GAA)_≥250_. Among the 476 individuals with undiagnosed ataxia, (GAA)_≥250_ was observed in six (1.3%); this frequency was higher than that in healthy individuals (0.2%). The clinical characteristics of the MSA patient with (GAA)_≥250_ were consistent with those of MSA, but not with SCA27B. Further research is warranted to explore the possibility of the potential association of expanded GAA repeats in *FGF14* with MSA.

## Introduction

Multiple system atrophy (MSA) is a progressive neurodegenerative disease characterized by autonomic failure, parkinsonism, cerebellar ataxia, and pyramidal features in various combinations [1]. Its diagnosis requires the onset of symptoms in individuals aged 30 or above, with autonomic dysfunction, along with cerebellar ataxia (MSA-C) or poorly l-dopa-responsive parkinsonism (MSA-P) [2, 3]. Although MSA has been generally considered a sporadic, nongenetic disease, several multiplex families with MSA have been reported, and *COQ2* and *GBA1* have been reported to be associated with MSA, raising a possibility that a genetic background underlies MSA [4, 5].

Spinocerebellar ataxia type 27B (SCA27B, MIM: 620174) is a recently established late-onset cerebellar ataxia associated with a pure GAA repeat expansion exceeding 250 repeat units in intron 1 of the fibroblast growth factor 14 gene (*FGF14*) [6–14]. Although the mode of inheritance of SCA27B is autosomal dominant, the penetrance is not complete, and some patients appear sporadic. Symptoms typically manifest as cerebellar ataxia, but cases with additional autonomic nervous system dysfunction are also known [11, 14]. Thus, there is a possibility of overlaps in the clinical presentations between SCA27B and MSA. In one study, three of 24 patients diagnosed as having possible MSA-C according to Gilman’s consensus criteria had repeat expansions with (GAA)_≥250_ in *FGF14* [15].

In this study, we searched for expanded GAA repeats in intron 1 of *FGF14* in 548 MSA patients from the MSA registry in Japan [16]. We further searched for expanded GAA repeats in patients with undiagnosed ataxia and healthy individuals, and compared the frequencies of expanded repeats among the groups. A previous report has shown two patients with ataxia carrying expanded pure GAA repeats exceeding 250 repeat units embedded in complex configurations, raising the possibility that such repeat configurations are also involved in SCA27B [17]. With this as a background, we conducted a detailed analysis of repeat configurations by the long-read sequence analysis of long-range polymerase chain reaction (LR-PCR) products containing expanded GAA repeat regions in *FGF14*.

## Materials and methods

### Study population

We recruited 548 Japanese patients with MSA registered in the Japan MSA Registry (https://msajp.org/) [16, 18, 19]. The diagnosis of MSA was made on the basis of Gilman’s consensus criteria, fulfilling possible or probable MSA [2]. The ages at onset of the MSA patients were 57.0 ± 8.9 years [mean ± standard deviation (SD); range, 37–76] for males and 58.6 ± 9.1 years (mean ± SD; range, 39–84) for females. The number of patients with MSA-C was 349 (150 males and 199 females), whereas that of patients with MSA-P was 181 (104 males and 77 females). The disease subtypes of 19 patients were unclassifiable on the basis of Gilman’s consensus criteria.

A total of 476 Japanese patients presenting with cerebellar ataxia of unknown etiologies were also included in the analysis of intronic GAA repeats in *FGF14*, with the following inclusion criteria: (i) progressive ataxia; (ii) onset after the age of 20 years; (iii) exclusion of secondary ataxias due to cerebrovascular diseases, tumor, alcohol abuse, autoimmune diseases, endocrinological diseases, or remote effects of malignancies; (iv) negative for SCAs 1, 2, 6, 7, 8, 12, 17, 31, and 36, Machado–Joseph disease (SCA3), and dentatorubral-pallidoluysian atrophy. A family history of cerebellar ataxia in first-degree relatives was identified in 58 patients (27 males and 31 females), and 418 patients were diagnosed as having sporadic cerebellar ataxia (233 males, 184 females, and one individual with no sex information).

A total of 455 healthy individuals (238 males, 199 females, and 18 individuals with no sex information) were included in the study. Of these, 45 were recruited from the University of Tokyo Hospital, 238 were obtained from the Japan Biological Informatics Consortium, and 172 were from the Japan Multiple System Atrophy Research Consortium [20]. The age of the healthy individuals when blood samples were obtained was 47.8 ± 16.0 years (mean ± SD).

Peripheral blood samples were obtained with written informed consent. This study was approved by the institutional review board of the University of Tokyo and participating institutions.

### Molecular genetic analysis

**Fig. 1:**
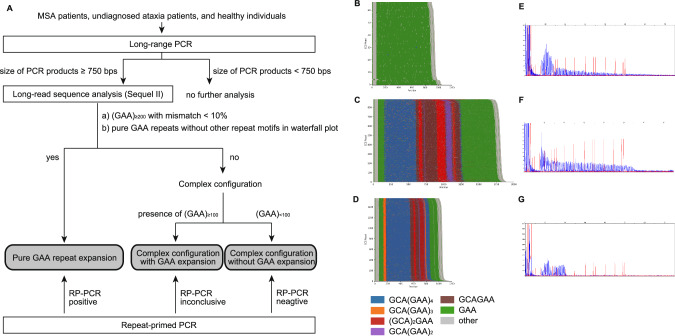
Analysis of expanded GAA repeats in the intron 1 of *FGF14.* **A** Flowchart of analysis of repeat configurations. **B** Representative waterfall plot of a patient with MSA who had an expanded repeat consisting of pure GAA repeats. Pure GAA repeats are shown in green. Note that a substantial number of mismatched nucleotides are observed in the reads derived from pure expanded GAA repeats possibly reflecting errors in the LR-PCR or in the long-read sequence analysis, which are more numerous than those observed in (**C**) or (**D**). **C** Waterfall plot of a patient with ataxia who had a complex configuration containing GAA repeats with 100–200 repeat units. **D** Waterfall plot of a patient with ataxia who had a complex configuration with GAA repeat units less than 100 repeat units. **E**–**G** Fragment analysis of the RP-PCR products of the same patients as shown in Fig. 1B–D. The result of the RP-PCR was interpreted as positive (**E**), inconclusive (**F**), and negative (**G**). RP-PCR, repeat-primed PCR.

The analytic flow, as shown in Fig. 1A, outlines the process for amplifying and analyzing the GAA repeat region in *FGF14* by LR-PCR, repeat-primed PCR (RP-PCR), and long-read sequencing. Briefly, the GAA repeat region in *FGF14* was amplified by LR-PCR as previously described [7, 10]. The LR-PCR products were electrophoresed in 1% agarose gels for 30 min at 100 V. Since previous reports showed that GAA repeats exceeding 200 repeat units may be associated with SCA27B [7], we selected the LR-PCR products with more than 200 repeat units to be analyzed. Since the size of the region containing the 200 repeat units ant the 150 bps flanking sequences is estimated to be 750 bps, the LR-PCR products showing mobilities of more than 750 bps on the agarose gel were excised and purified using a QIAquick Gel Extraction Kit (QIAGEN, California, USA; Supplementary Fig. 1A). For the five individuals in whom no LR-PCR products with sizes smaller than 750 bps were observed, the reaction solutions containing the LR-PCR products were directly purified using a QIAquick Gel Extraction Kit skipping the preparative agarose gel electrophoresis (Supplementary Fig. 2A). The purified LR-PCR products were then subjected to long-read sequence analysis using SMRTbell barcoded adapter plate 3.0, SMRTbell prep kit 3.0, and Sequel II binding kit 3.2 (Pacific Biosciences, CA, USA) according to the manufacturer’s instructions. RP-PCR analysis targeting the expanded GAA repeats was performed as previously described [7, 10].

Since the number of reads with expanded repeats tended to be less, we conducted long read sequence analysis using two flow cells for individuals with insufficient reads obtained using one flow cell.

### Determination of the configuration of expanded repeats employing long-read sequence analysis

To determine the size and configuration of expanded repeats in the reads obtained by long-read sequence analysis of LR-PCR products, the HiFi reads containing the GAA repeat regions of *FGF14* were extracted for detailed analysis of repeat configurations. Considering the possibility of errors derived from the long-read sequence analysis of the LR-PCR products in some of the reads, fastq files were searched using the non-overlapping unique sequences flanking the GAA repeat region. The three sequences upstream of the GAA repeats were 5′-TGCAAATGAAGGAAAACTCTTA, 5′-TCTTAGTTGTAAAATATCAATA, and 5′-TTCTCTATGCAACCAACTTT and those downstream of the GAA repeats were 5′-GAAATGTGTTTAAGAATTCCTCAA, 5′-TAAGACTAAGCTCTATGTGGG, and 5′-CAGGAACTGCTTAATTCATCATTG (Supplementary Fig. 1C). Since we occasionally experience read errors in the long-read sequence analysis, the reads containing at least one of the three sequences on both sides were extracted for further analysis. The repeat configurations of the extracted reads were visualized using waterfall.py (Pacific Biosciences, https://github.com/PacificBiosciences/apps-scripts/tree/master/RepeatAnalysisTools) with six repeat motifs: GCA(GAA)_4_, GCA(GAA)_3_, (GCA)_2_GAA, GCA(GAA)_2_, GCAGAA, and GAA. The repeat configurations were determined on the basis of the waterfall plots. Since the extracted reads occasionally showed a broad length distribution, we identified a region displaying uniform read lengths in the waterfall plot, and the reads within this region were extracted and the sizes of expanded repeats were determined (Supplementary Figs. 1B and 3). This region is considered suitable for the analysis of expanded repeats and was extracted for further analysis. The sizes of these extracted reads closely matched the sizes estimated by the agarose gel electrophoresis (Supplementary Table 1).

### Analyses of long-read whole genome sequence results

Samples were prepared using SMRTbell prep kit 3.0 and Sequel II binding kit 3.2 (Pacific Biosciences, CA, USA) according to the manufacturer’s instructions. Circular consensus sequencing (CCS) reads were aligned using minimap2 (https://github.com/lh3/minimap2) [21] with GRCh38 as the reference genome, and the reads containing the repeat region of *FGF14* were extracted.

### Determination of the number of repeat units employing long-read sequence analysis

Because long-read sequence data inevitably contains read errors with low complexity sequences such as expanded tandem repeats, we determined the size of expanded repeats based on the junction points between the repeat sequence and the unique sequences flanking the repeat sequence. Reads that contain the junction sequence (5′-AACCAACTTTCTGTGAAGAA for the upstream junction sequence and 5′-GAAGAATAGAAATGTGTTTA for the downstream junction sequence) were identified, and the length between the junction points was calculated as the number of the repeat units. Employing this method, we determined the sizes of expanded GAA repeats in the reads obtained from the LR-PCR products as well as those in the reads obtained from the PCR-free long-read whole genome sequence analysis.

## Results

### Configurations of expanded repeats in intron 1 of *FGF14*

We first conducted LR-PCR analysis to identify samples that showed LR-PCR products derived from intron 1 of *FGF14* with sizes >750 bps in patients with MSA, patients with undiagnosed ataxia, and healthy individuals (Fig. 1, Table 1). LR-PCR products with the size >750 bp were observed in 36 patients with MSA (6.6%), 24 patients with undiagnosed ataxia (5.0%), and 28 healthy individuals (6.2%), indicating no differences among these groups. Considering the possibility that repeat configurations are different in these individuals, we then analyzed the repeat configurations of these LR-PCR products in detail by the long-read sequence analysis. The median number of reads obtained per individual was 1644 (range, 22–17,480). We first generated a waterfall plot to obtain an overview of the entire repeat regions. The repeat configurations were classified into three categories: (1) expanded repeats consisting of pure GAA repeats (Fig. 1B, Supplementary Fig. 3), (2) complex configuration with GAA repeats with the number of repeat units exceeding 100 (Fig. 1C), and (3) complex configuration with GAA repeats less than 100 repeat units (Fig. 1D). The frequently observed repeat motifs other than pure GAA were GCA(GAA)_4_, GCA(GAA)_3_, (GCA)_2_GAA, GCA(GAA)_2_, and GCAGAA.

**Table 1:** Frequencies of expanded repeats consisting of pure GAA, complex configuration with pure GAA expansion, and complex configuration without pure GAA expansion in patients with undiagnosed ataxia, those with MSA, and healthy individuals.

	*N*	LR-PCR positive	Pure GAA expansion (GAA) ≥ 250	Pure GAA expansion (GAA)200-249	Pure GAA expansion (GAA) < 200	Complex configuration with pure GAA expansion	Complex configuration without pure GAA expansion
Undiagnosed ataxia (GAA repeat units)	476	24 (5.0%)	6 (1.3%) (251–361)	2 (0.4%) (212–240)	1 (0.2%) (191)	1 (0.2%) (167)	14 (2.9%) (24–83)
MSA (GAA repeat units)	548	36 (6.6%)	1 (0.2%) (269)	0	0	1 (0.2%) (134)	34 (6.2%) (21–83)
Healthy individuals (GAA repeat units)	455	28 (6.2%)	1 (0.2%) (254)	1 (0.2%) (245)	0	2 (0.4%) (140–160)	24 (5.3%) (23–75)

The range of the number of GAA repeat units of each category is shown in parentheses.

*LR-PCR* long-range PCR, *MSA* multiple system atrophy.

Expanded repeats consisting of pure GAA repeats exceeding 250 repeat units, which correspond to the full penetrance or incomplete penetrance ranges of SCA27B, as reported in a previous study [7], were observed in six patients with undiagnosed ataxia (1.3%), one patient with MSA (0.2%), and one healthy individual (0.2%) (Table 1). All individuals with pure GAA repeat expansion were positive in RP-PCR (Fig. 1E). Although the pathogenicity for SCA27B is unknown, GAA repeats with 200–249 repeat units were observed in two patients with undiagnosed ataxia (0.4%), and one healthy individual (0.2%), whereas those repeats were not observed in patients with MSA. In addition, one patient with undiagnosed ataxia (0.2%) had GAA repeats with 191 repeat units.

The complex configurations containing GAA repeats with 100–200 repeat units were observed in one patient with undiagnosed ataxia (0.2%), one patient with MSA (0.2%), and two healthy individuals (0.4%). The RP-PCR signals in individuals with the complex configuration containing GAA repeats with 100–200 repeat units were initially positive but weakened and disappeared halfway of the capillary electrophoresis analysis, which was defined as an “inconclusive RP-PCR result” (Fig. 1F). The complex configuration with GAA repeats less than 100 repeat units was observed in 14 patients with ataxia (2.9%), 34 patients with MSA (6.2%), and 24 healthy individuals (5.2%). The individuals who had the complex configuration with GAA repeats less than 100 repeat units were negative in RP-PCR (Fig. 1G).

### Size distribution and frequency of expanded GAA repeats

**Fig. 2:**
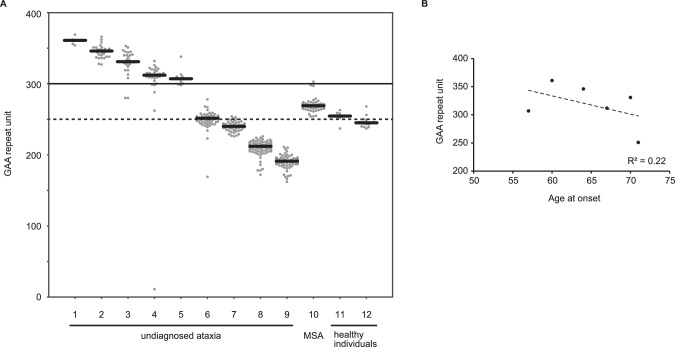
Size distribution and frequency of expanded GAA repeats in the patients with ataxia, patients with MSA, and healthy individuals. **A** Swarm plots showing the number of the GAA repeat units for each read in patients with ataxia (*n* = 9), a patient with MSA (*n* = 1), and healthy individuals (*n* = 2) who had expanded repeats consisting of pure GAA repeats. Short black line in each individual showed median of the repeat units. The horizontal bold black line indicates the 300 GAA repeat units. The GAA repeat units exceeding 300 are considered to have full penetrance. The dashed black line indicates the 250 GAA repeat units, indicating that GAA repeats ranging from 250 to 299 repeat units are considered to have incomplete penetrance. **B** An inverse correlation between age at onset of SCA27B and the number of GAA repeat units of *FGF14* (*n* = 6). The dashed line shows the linear regression.

Next, we investigated the size and frequency of expanded pure GAA repeats (Fig. 2A, Table 1). We found that six (1.3%) patients with undiagnosed ataxia had expanded pure GAA repeats with (GAA)_≥250_, which were more frequent than those in healthy individuals (0.2%) with expanded pure GAA repeats (*p* = 0.18, Fisher’s exact test). Of these six patients with undiagnosed ataxia, five had (GAA)_≥300_, which was interpreted as the full penetrance range according to a previous report, and the other one had (GAA)_250–299_, which was considered within the incomplete penetrance range [7]. One MSA patient (0.2%) had expanded pure GAA repeats of 269 repeat units. One healthy individual had expanded pure GAA repeats of (GAA)_250–299_, whereas neither MSA patients nor healthy individuals had (GAA)_≥300_.

### Comparison of the size of expanded GAA repeats determined by the long-read sequence analysis of the LR-PCR products and that determined by the PCR-free whole genome long-read sequence analysis

We performed the PCR-free long-read whole genome sequence (WGS) analysis of genomic DNAs obtained from two individuals, to compare with the sizes determined by the long-read sequences of the LR-PCR products. One was a patient with MSA who had a pure GAA repeat expansion (Fig. 1B), and the other was a patient with undiagnosed ataxia who had a pure GAA repeat expansion and a complex repeat configuration as a compound heterozygous state (the same patient as shown in Supplementary Fig. 2). Based on the junctions of the GAA repeats and the flanking sequence in the CCS reads, we determined the length between the junction points as the size of expanded GAA repeats. In the PCR-free long-read WGS analysis data, there were three reads from the patient with MSA and five reads from the patient with undiagnosed ataxia (Supplementary Fig. 4). The median repeat units determined by the PCR-free long-read WGS analysis were 283 (range, 282–285) in the patient with MSA and 318 (range, 317–325) in the patient with undiagnosed ataxia. The median repeat units estimated by the long-read sequence analysis of the LR-PCR products were 269 and 307 in the MSA patient and the undiagnosed ataxia patient, respectively, which were slightly shorter than those determined by the PCR-free long-read WGS analysis (Supplementary Fig. 4C).

### Clinical characteristics of patients with ataxia with expanded pure GAA repeats

The clinical characteristics of the six patients with undiagnosed ataxia carrying (GAA)_≥250_ are summarized in Table 2. Although the clinical significance of (GAA)_200–249_ is not clearly established, the clinical presentations of the two ataxia patients carrying (GAA)_200–249_ are also included in Table 2. The age at onset and the age at examination were 64.8 ± 5.1 years (mean ± SD; range, 57–71) and 71.7 ± 5.7 years (mean ± SD; range, 64–78), respectively. The number of GAA repeat units and the age at onset tended to be inversely correlated, although this was not statistically significant (Fig. 2B, Spearman’s *r* = −0.37, *p* = 0.50).

**Table 2:** Clinical characteristics of patients with undiagnosed ataxia carrying (GAA) ≥ 250 or ataxia carrying (GAA)200_–_249 and patients with MSA carrying (GAA) ≥ 250.

	Ataxia (GAA) ≥ 250	Ataxia (GAA) 200-249	MSA (GAA) ≥ 250
*N*	6	2	1
Clinical diagnosis when referred to genetic diagnosis	SCD 4, CCA 2	SCD 2	MSA-C^a^
Family history	1 (16.7%)	1 (50%)	0
Mode of inheritance**	AD 1/1 (100%)	AD 1/1 (100%)	NA
Number of male patients	4 (66.7%)	1 (50%)	1 (100%)
Age at onset	65.5 (57–71)	51.5 (25–78)	59
Age at examination	74.0 (64–78)	54.0 (30–78)	67
Episodic symptoms	2/5 (40%)	0/1 (100%)	NA
Saccadic pursuit of eye movement	5/5 (100%)	1/1 (100%)	NA
Downbeat gaze nystagmus	1/4 (25%)	0/2 (0%)	NA
Horizontal gaze nystagmus	3/5 (60%)	1/2 (50%)	NA
Oscillopsia	2/5 (40%)	0/1 (0%)	NA
Cerebellar dysarthria	4/6 (66.7%)	2/2 (100%)	NA
Dysphagia	3/6 (50%)	0/2 (0%)	1 (100%)
Gastrostomy	0/6 (0%)	0/2 (0%)	1 (100%)
Appendicular ataxia	5/6 (83.3%)	2/2 (100%)	1 (100%)
Truncal ataxia	6/6 (100%)	2/2 (100%)	1 (100%)
Ataxic gait	4/5 (80%)	2/2 (100%)	1 (100%)
Unable to walk	0/6 (0%)	0/2 (0%)	1 (100%)
Positive Babinski signs	0/4 (0%)	0/1 (0%)	0 (0%)
Postural tremor	2/6 (33.3%)	0/2 (0%)	NA
Orthostatic hypotension	0/2 (0%)	0/2 (0%)	1 (100%)
Urinary incontinence	0/6 (0%)	0/2 (0%)	1 (100%)
Residual urine	0/6 (0%)	0/2 (0%)	1 (100%)
Urinary catheter	0/6 (0%)	0/2 (0%)	1 (100%)
NPPV	0/6 (0%)	0/2 (0%)	1 (100%)
Cerebellar atrophy (MRI)	3/5 (60%)	2/2 (100%)	1 (100%)
Vermis atrophy (MRI)	3/5 (60%)	1/2 (50%)	NA
Pontine atrophy (MRI)	0/5 (0%)	0/2 (0%)	1 (100%)
Cerebellar hypoperfusion on SPECT	1/3 (33.3%)	1/1 (100%)	NA

When sufficient clinical information is unavailable for particular items, they are not included in the table.

*AD* autosomal dominant inheritance, *NPPV* non-invasive positive pressure ventilation, *SPECT* ^123^I-IMP single-photon emission computed tomography, *SCD* spinocerebellar degeneration, *MSA* multiple system atrophy, *MSA-C* multiple system atrophy, cerebellar type, *CCA* cortical cerebellar atrophy, *NA* not available

^a^Possible MSA-C based on Gilman’s consensus criteria.

**Mode of inheritance has been described in patients with family history.

**Fig. 3:**
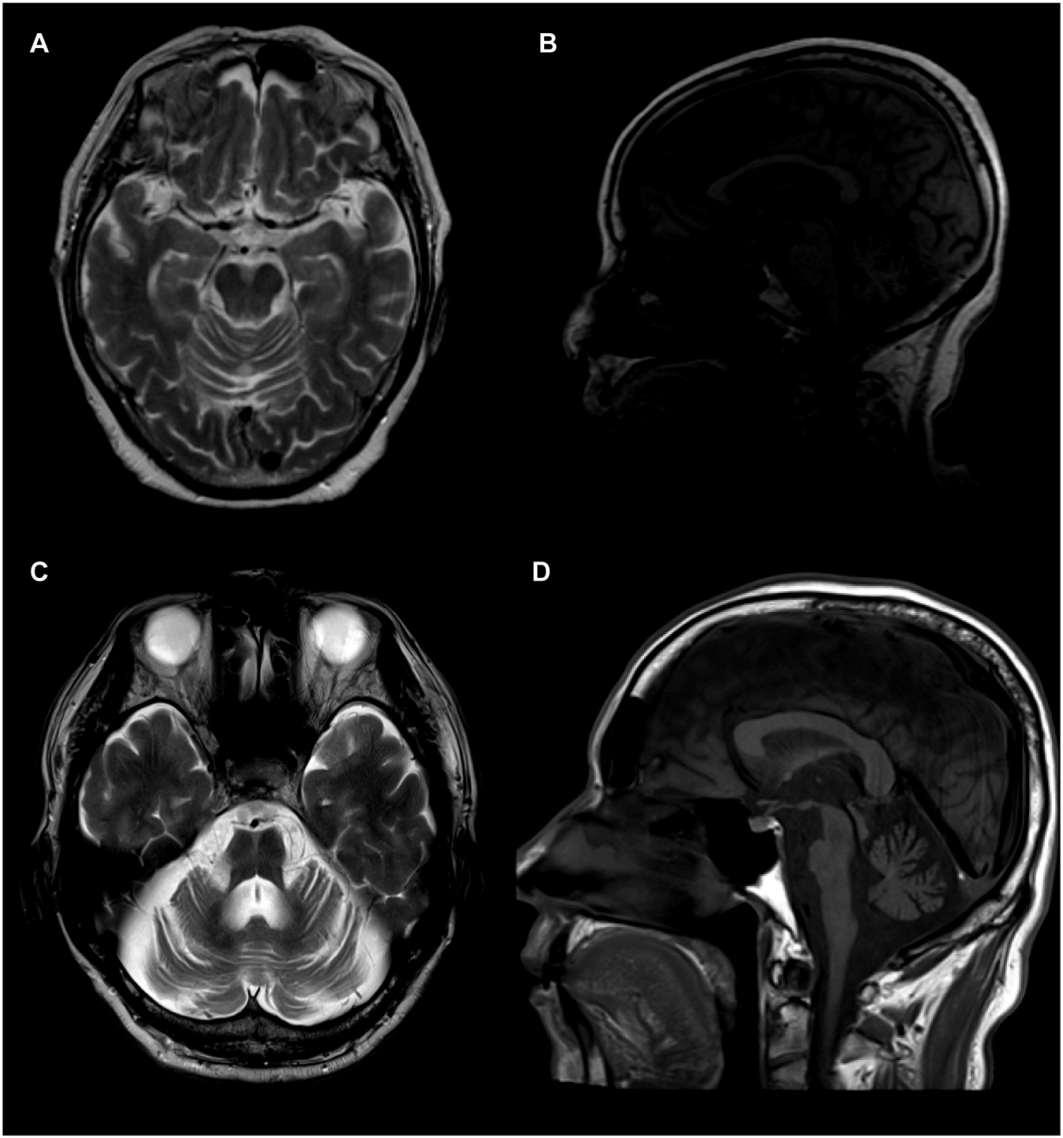
Brain MRI of the patients with MSA or undiagnosed ataxia with expanded GAA repeats. Axial T2-weighted image (**A**) and sagittal T1-weighted image (**B**) of the patient with undiagnosed ataxia who had (GAA)_312_. MRI was performed at the age of 84, twelve years after the symptom onset. Atrophic changes in the upper part of the cerebellar hemisphere and the vermis were observed. The brainstem was not atrophic. Axial T2-weighted image (**C**) and sagittal T1-weighted image (**D**) of the patient with MSA who had (GAA)_269_ seven years after the symptom onset. Atrophic changes in the cerebellum, middle cerebellar peduncles, and basis pontis were observed. The hot cross bun sign was observed in (**C**).

Of the six ataxia patients with (GAA)_≥250_, four patients were previously diagnosed as having spinocerebellar degeneration, and the other two were previously diagnosed as having cortical cerebellar ataxia. The one patient (16.7%) had a family history of ataxia, which is consistent with the autosomal dominant mode of inheritance. Episodic symptoms, including blurred vision or slurring of speech during prolonged physical activity, were observed in two patients as their initial clinical manifestations (40%). One patient reported experiencing a blurred vision of the tennis ball after playing tennis for 20 to 30 min. The other patient exhibited slurred speech resembling drunkenness during prolonged conversation and reported feeling unsteady when walking for seven kilometers. Gaze-evoked downbeat nystagmus was observed in one (25%) patient and horizontal nystagmus in three (60%) patients. Oscillopsia was observed in two patients (40%). On brain MRI, atrophic changes in the hemisphere and vermis of the cerebellum were observed in three patients with undiagnosed ataxia carrying (GAA) _≥250_ (60%) (Fig. 3A, B). None of the patients were unable to walk, had gastrostomy due to dysphagia, or had noninvasive positive pressure ventilation (NPPV). No extrapyramidal signs were documented.

### Clinical characteristics of a patient with MSA with expanded pure GAA repeats

The clinical characteristics of one patient with MSA carrying (GAA)_269_ are summarized in Table 2. There was no family history of MSA or ataxia. His symptom started at the age of 59, and he was registered in the Japan MSA Registry at 67. He had cerebellar ataxia, as well as urinary incontinence, residual urine, and orthostatic hypotension at registration. Brain MRI showed atrophic changes of the cerebellum, middle cerebellar peduncles, and basis pontis (Fig. 3C, D). At age 69, the patient became unable to walk even with assistance. At age 70, he began receiving NPPV. At age 71, a urinary catheter was placed, and gastrostomy was performed due to dysphagia. Neither downbeat nystagmus, oscillopsia, nor episodic ataxia/oscillopsia was observed in the patient.

### Pure GAA repeat expansion embedded in the complex configurations

Regarding the repeats with the complex configurations with GAA repeats with 100–200 repeat units observed in four individuals (one ataxia patient, one MSA patient, and two healthy individuals; Fig. 1C), the waterfall plots showed that pure GAA repeats were located at both ends of the complex configurations. The numbers of GAA repeat units at the upstream end, as defined by the transcription direction of *FGF14*, were 34 in one ataxia patient, 27 in one MSA patient, and 27 and 31 in two healthy individuals. The numbers of GAA repeat units at the downstream end were 167 in one ataxia patient, 134 in one MSA patient, and 160 and 140 in two healthy individuals.

### The single-nucleotide polymorphism rs534066520 is associated with complex repeat configuration

A recent study reported that the single-nucleotide polymorphism (SNP) rs534066520 (GRCh38, chr13:102161565 T>A) is observed in the allele with complex configurations carrying the (GCAGAA)n repeat expansions, while the wild-type genotype (T) is observed in wild-type alleles or in alleles with pure GAA repeat expansions.22 We confirmed that all the twelve individuals with expanded pure GAA repeats had the wild-type allele (T) of rs534066520, whereas individuals who had complex repeat configurations had the A allele of rs534066520. Regarding the 17 bp deletion-insertion variant in the 5′-flanking region (5′-CFV) that is reportedly associated with enhanced stability of the FGF14 repeat locus,23 there were no individuals carrying the 5′-CFV insertion in the present study.

## Discussion

We searched for expanded GAA repeats in intron 1 of *FGF14* in undiagnosed ataxia patients and patients with MSA in the Japanese population. (GAA)_≥250_ was observed in six undiagnosed ataxia patients (1.3%), which was more frequent than in healthy individuals (0.2%) and patients with MSA (0.2%). These observations are consistent with previous findings that pure expanded GAA repeats are involved in SCA27B [6–14].

There are populational differences in the frequency of expanded GAA repeats in patients with SCA27B and healthy individuals. The (GAA)_≥250_ repeat expansion was present in 61%, 18%, 15%, and 10% of French–Canadian, German, Australian, and Indian patients with undiagnosed ataxia, respectively [7]. In contrast to previous reports, the frequency of SCA27B in undiagnosed ataxia patients was relatively low with 1.3% in the Japanese population in this study. Previous reports from Japan also indicated low frequencies of SCA27B in the late-onset ataxia case series, with frequencies of 1.2% and 3.0% [9, 22]. Previous reports from China described that the frequencies of SCA27B was 1.3% and 0.3% in patients with late-onset ataxia [17, 23]. Thus, the frequencies of SCA27B are relatively low in East Asian regions. The frequency of expanded GAA repeats in healthy individuals was reported to be 1% in French–Canadians and 3% in Germans, whereas it was 0.2% in the Japanese population in this study [7]. The number of repeat units in the expanded GAA repeats in the Japanese healthy individuals ranged between 250 and 299 GAA in number, which is also consistent with previous studies [7, 10]. It may be interpreted that GAA repeat units ranging in number from 250 to 299 do not have full penetrance on ataxia onset [7, 10]. Another possibility is that the age of healthy individuals at examination was younger than that of ataxia patients, which may lead to the overestimation of the carrier frequency of expanded GAA repeats in Japan. The healthy individual with (GAA)_250–299_ repeat expansion was a 24-year-old man. Given the late onset of SCA27B, it is possible that the individual who was healthy at the time of examination may develop SCA27B in the future.

We observed that two patients with undiagnosed ataxia had expanded pure GAA repeats with the number of repeat units ranging from 200 to 249 (Tables 1 and 2). Although it remains elusive whether GAA repeat units ranging in number from 200 to 249 are associated with ataxia, a previous report documented a family of late-onset pure cerebellar ataxia, in which the patient and her affected mother had pure GAA repeat expansions with 256 repeat units and 234 repeat units, respectively [24]. To determine the lower limit of the number of GAA repeat units for the development of SCA27B, further investigation should be conducted to identify individuals with GAA repeats fewer than 250.

We further compared the number of repeat units obtained by the PCR-free long-read WGS analysis and that determined by the long-read sequence analysis of the LR-PCR products in the two patients. The number of repeat units of the LR-PCR products were estimated to be 14 to 11 units shorter compared with those determined by the PCR-free long-read WGS analysis. PCR artifacts presumably underlie the shorter repeat units of the LR-PCR products. Although we conducted the long-read WGS analysis of the two individuals, the observation raised the possibility that there are cases where the actual number of repeat units is underestimated when the repeat units are determined by the long-read sequence analysis of the LR-PCR products, leading to underdiagnosis of SCA27B. When the long-read sequence analysis of the LR-PCR products shows GAA repeat units close to or fewer than 250, PCR-free long-read WGS should also be considered.

We observed one MSA-C patient with (GAA)_≥250_. The frequency of expanded GAA repeats in the MSA group was similar to that in the healthy individuals (Table 1). Clinically, the MSA patient with (GAA)_≥250_ presented with cerebellar symptoms and autonomic dysfunction, and later became unable to walk, requiring NPPV and gastrostomy. Brain MRI of the patient showed atrophic changes in the cerebellum, pons, and middle cerebellar peduncles. These findings were consistent with the natural history and clinical characteristics of MSA and differed from the clinical presentation of SCA27B [16, 20]. The frequency of individuals carrying pure GAA repeat expansions with the number of repeat units exceeding 250 was similar between the MSA and control groups, suggesting that the MSA patient could incidentally be a carrier of the pure GAA repeat expansion. To further explore the potential involvement of expanded GAA repeats in MSA, analysis of much larger number of patients with MSA will be needed.

We also found that pure GAA repeats in the complex configuration located downstream of *FGF14* in the transcription direction were occasionally long (Fig. 1C). Because the pure GAA repeat expansion is considered important for the development of ataxia symptoms, it is necessary to evaluate the pure GAA repeats located downstream of the repeat region embedded in the complex configuration [7, 17, 25]. However, when RP-PCR positivity or the size determination of the LR-PCR products is employed, there is a risk of overestimating the expanded GAA repeats in analyzing expanded GAA repeats embedded in the complex configurations. To accurately determine the number of repeat units in such individuals, long-read sequence analysis, as we conducted in this study, would be essential. In this study, we did not find any individuals with a complex configuration with expanded GAA repeats exceeding 250 repeat units. However, a previous report has documented patients in whom downstream pure GAA repeats exceeded 250 repeat units and presented with ataxia [17].

Several limitations need to be considered in our present study. First, LR-PCR may have introduced some errors in the repeat regions. Indeed, although the LR-PCR products were gel-electrophoresed and then excised from the gel at their specific mobilities, we still observed a heterogeneity in the sizes of the obtained HiFi reads (Supplementary Figs. 1 and 2). It cannot be ruled out that LR-PCR and long-read sequence analysis have introduced some read errors. We examined the waterfall plot and fastq file for each individual and confirmed that expanded pure GAA repeat sequences could be unambiguously discriminated from repeat sequences with regularly appearing interrupted repeat configurations (Fig. 1). Another limitation is that the group of patients with undiagnosed cerebellar ataxia may comprise heterogeneous etiologies. In addition, the clinical information of patients with undiagnosed ataxia was not fully available since the majority of them were referred to our laboratory for molecular diagnosis from other hospitals.

## Conclusion

In this study, we confirmed the presence of pure GAA repeat expansions in 1.3% of the undiagnosed ataxia patients in Japan, supporting the diagnosis of SCA27B. The clinical manifestations of SCA27B in the Japanese population were similar to those documented previously, characterized by late-onset ataxia, downbeat nystagmus, episodic symptoms, and atrophic cerebellar vermis on MRI. Of the 548 MSA patients, we identified only one with expanded GAA repeats, who presented typical clinical features of MSA. Further research is warranted to explore the possibility of the potential association of expanded GAA repeats in *FGF14* with MSA.

## Supplementary information

Supplementary Materials

## Data Availability

The data supporting the study are available from the corresponding author upon reasonable request.
