# Supplementary material for: Frequency of FGF14 intronic GAA repeat expansion in patients with multiple system atrophy and undiagnosed ataxia in the Japanese population
Source: Eur J Hum Genet. 2024 Nov 27;33(3):325–33. doi: 10.1038/s41431-024-01743-3 (PMC11893785; doi:10.1038/s41431-024-01743-3)
Supplement: Supplementary file 1 — Supplementary Materials [file 41431_2024_1743_MOESM1_ESM.pdf]

A

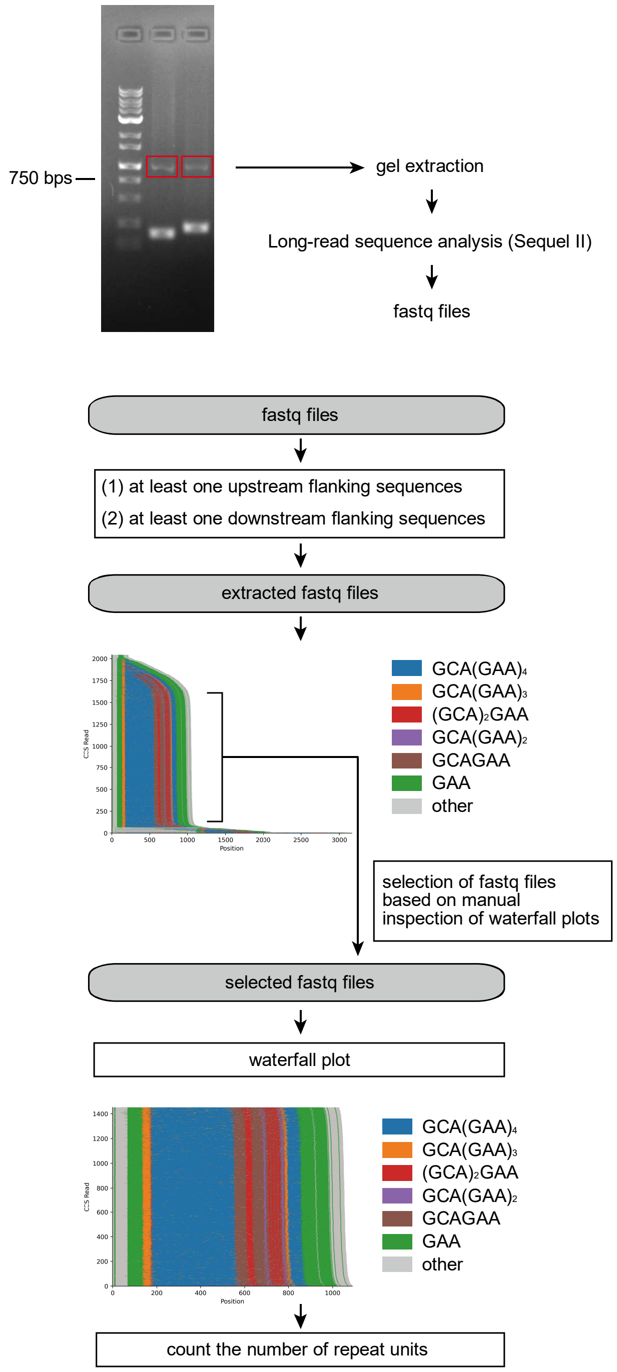

C

...TGCAAATGAAGGAAAACTCTTATCTTAGTTGTAAAATATCAATA
TTCTCTATGCAACCAACTTTCTGTGAAGAAAGAAA $(GAA)_{50}$ TA
GAAATGTGTTTAAGAATTCCTCAATAAGACTAAGCTCTATGTGGGCA
GGAACTGCTTAATTCATCATTG...

upstream flanking sequences:
- TGCAAATGAAGGAAAACTCTTA
- TCTTAGTTGTAAAATATCAATA
- TTCTCTATGCAACCAACTTT

downstream flanking sequences:
- GAAATGTGTTTAAGAATTCCTCAA
- TAAGACTAAGCTCTATGTGGGCA
- GGAACTGCTTAATTCATCATTG

Supplementary Figure 1. Data analysis of cases, where both large (≥ 750 bps) and small (< 750 bps) bands were observed in the agarose gel electrophoresis of the LR-PCR products.

(A) A representative image of an agarose gel electrophoresis. Samples with bands outlined by red lines (> 750 bps) are determined to be LR-PCR positive. These bands are extracted from the gel, and subjected to long-read sequence analysis. (B) To obtain the reads derived from the intron 1 of the *FGF14* gene, the reads containing at least one of the three flanking sequences at both sides were extracted as the "extracted fastq files". Since the reads in the "extracted fastq files" occasionally showed a broad length distribution, we identified a region displaying uniform read lengths in the waterfall plot, and the reads within this region were extracted as the "selected fastq files". Waterfall plot was produced with six repeat motifs, $GCA(GAA)_4$, $GCA(GAA)_3$, $(GCA)_2GAA$, $GCA(GAA)_2$, GCAGAA, and GAA. (C) GRCh38/hg38 reference genome sequence of the intron 1 in *FGF14*. The three upstream flanking sequences were derived from sequences flanking the GAA repeat regions upstream of the transcriptional direction of *FGF14*. The downstream flanking sequences were derived from the sequences flanking the GAA repeat regions downstream of the transcriptional direction of *FGF14*.

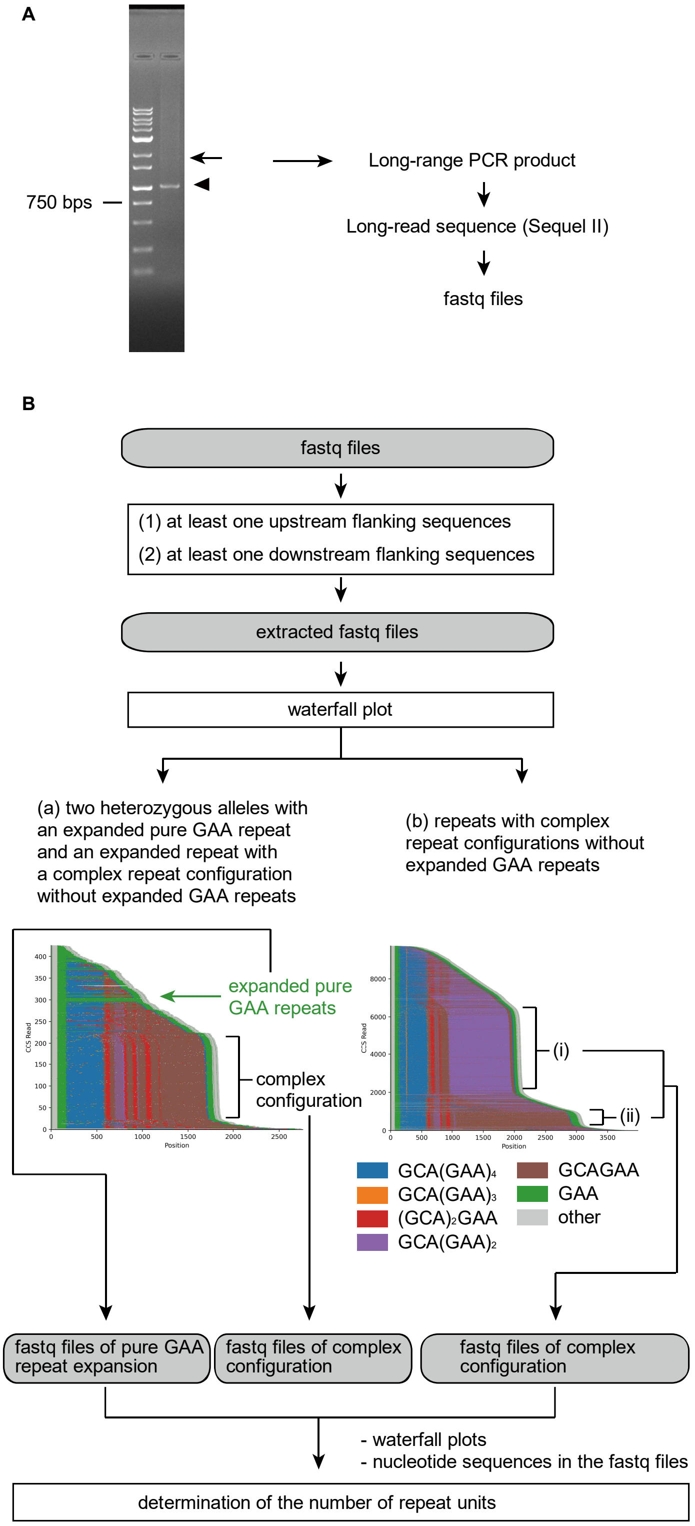

Supplementary Figure 2. Data analysis flow of the case, where short (< 750bp) bands of the LR-PCR products were not observed in the agarose gel electrophoresis.

(A) A representative image of the agarose gel electrophoresis without bands shorter than 750 bps.

There was a distinct band at around 1 kb (arrowhead), and a faint band visible around 1.8 kb (arrow). In these cases, the reaction solutions containing the LR-PCR products were directly purified using QIAquick Gel Extraction Kit skipping the preparative agarose gel electrophoresis and then subjected to the long-read sequence analysis. (B) When there are no short (< 750 bps) bands in the LR-PCR, waterfall plots are expected to show (1) expanded pure GAA repeats, (2) complex configuration with expanded pure GAA repeats, or (3) complex configuration without pure GAA repeats. The waterfall plot on the left panel shows two heterozygous repeat alleles with an expanded pure GAA repeat and a complex repeat configuration without expanded GAA repeats. The waterfall plot on the right panel shows two alleles of complex repeat configurations without expanded GAA repeats. It is of note that a small number of reads containing expanded repeats consisting of green-colored pure GAA repeats are observed in the reads with complex repeat configurations without expanded GAA repeats. Reads with pure GAA repeat expansion and those with complex configuration were extracted based on the visual inspection of the waterfall plots, and were further analyzed to determine the number of repeat units Waterfall plot of the “extracted fastq files” was produced using the waterfall.py with six repeat motifs, $GCA(GAA)_4$, $GCA(GAA)_3$, $(GCA)_2GAA$, $GCA(GAA)_2$, GCAGAA, and GAA.

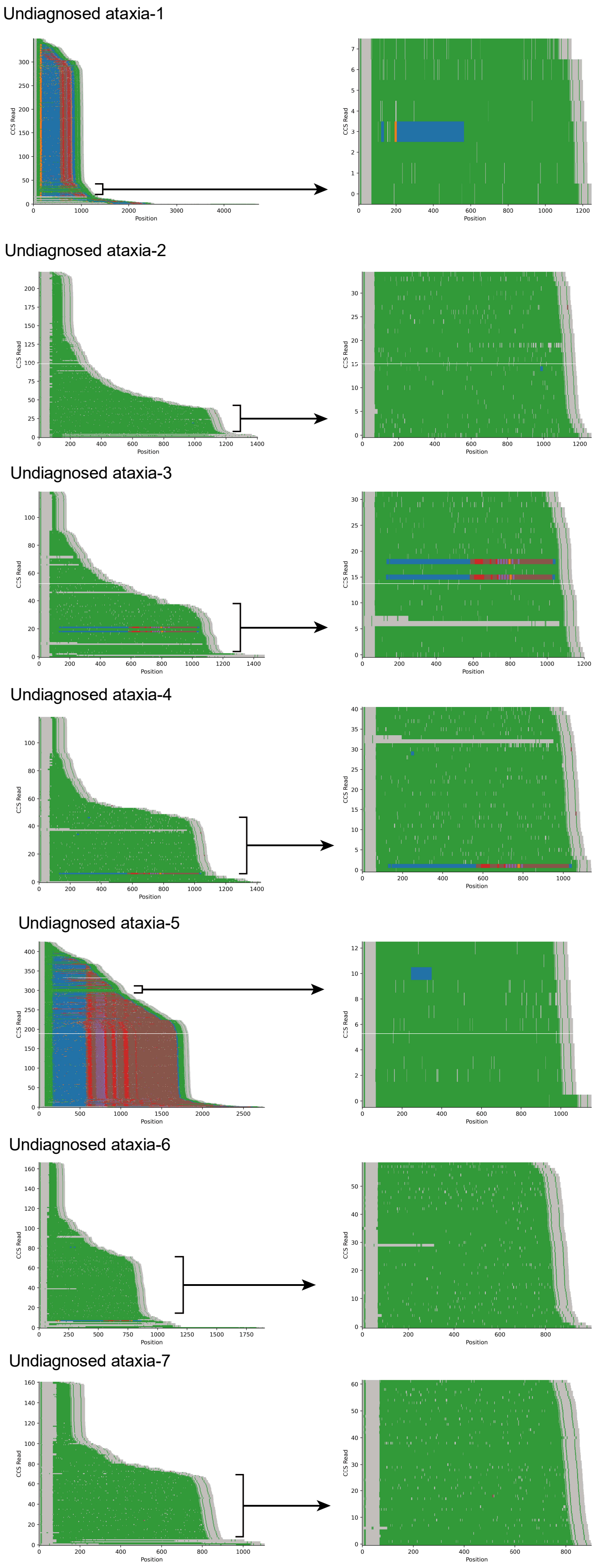
Undiagnosed ataxia-1
Undiagnosed ataxia-2
Undiagnosed ataxia-3
Undiagnosed ataxia-4
Undiagnosed ataxia-5
Undiagnosed ataxia-6
Undiagnosed ataxia-7
CCS Read
Position

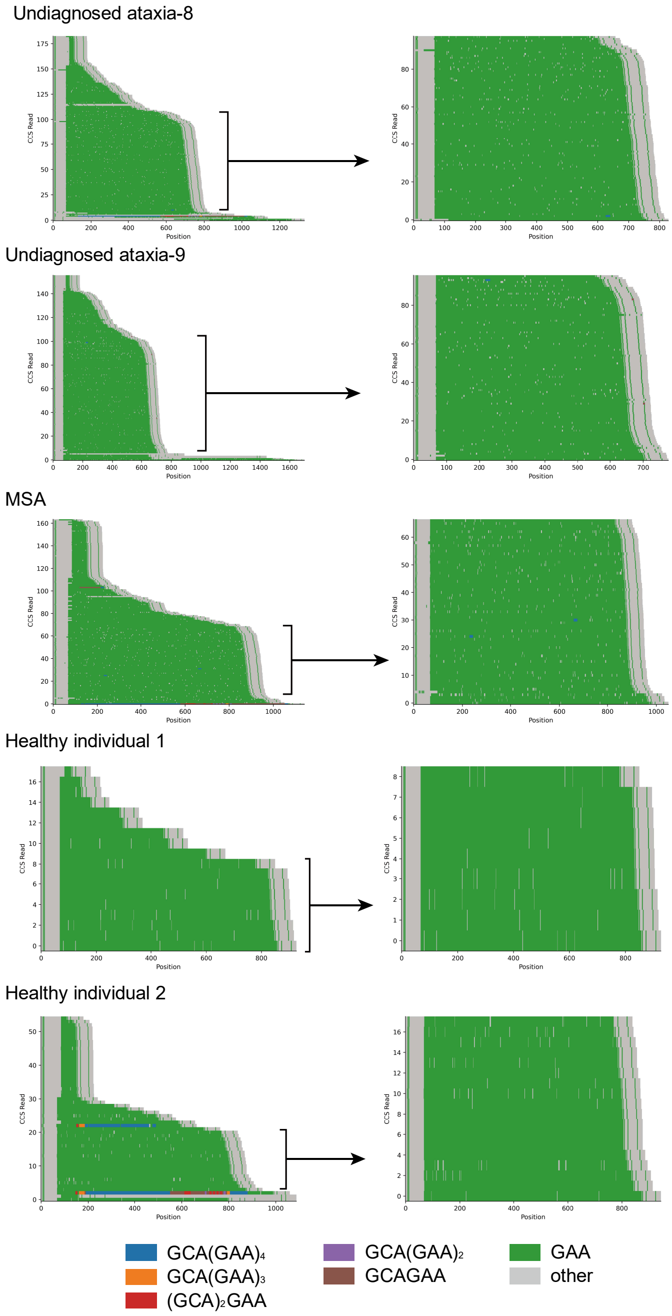

Supplementary Figure 3. Waterfall plots of the individuals carrying pure GAA repeat expansions. In each individual, left panel shows all the reads obtained from the long-read sequence analysis of LR-PCR products. In the left panel, a region displaying uniform read lengths was identified in the waterfall plot, and the reads within this region were extracted and displayed in the right panel. When LR-PCR did not produce bands shorter than 750 bps in the patients identified as Undiagnosed ataxia 1 and 6, the LR-PCR products were directly analyzed in Sequel II without gel extraction.

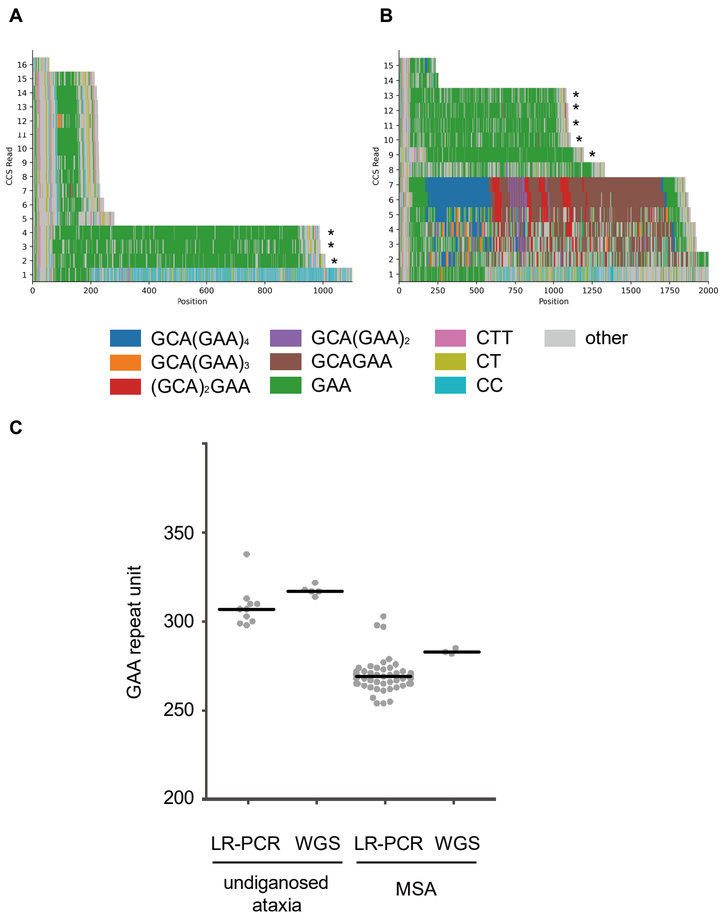

Supplementary Figure 4. Long-read, whole genome sequence (WGS) analysis of the two patients with pure GAA repeat expansion. (A, B) Waterfall plot of CCS reads obtained by whole genome sequencing analysis of *FGF14* repeat region of a patient with MSA (A) or a patient with undiagnosed ataxia (B). CCS reads containing pure GAA repeat expansion and both upstream and downstream flanking sequences (marked with asterisks) were further analyzed. (C) Comparison of the distribution of the number of GAA repeat units between WGS and LR-PCR-based analysis. Black lines indicate median of the number of repeat units. Note that reads obtained by long-read sequence analysis of LR-PCR products showed considerable variation, presumably reflecting PCR errors.

Supplementary Table 1. Comparison of length of the repeat region of *FGF14* estimated from long-read sequence analysis of LR-PCR product to that estimated from agarose gel electrophoresis.

	Length of the repeat region estimated from agarose gel electrophoresis (bps)	Length of the repeat region estimated from long-read sequence analysis (bps)
Undiagnosed ataxia 1	1050	1085
Undiagnosed ataxia 2	1050	1038
Undiagnosed ataxia 3	950	994
Undiagnosed ataxia 4	950	938
Undiagnosed ataxia 5	950	923
Undiagnosed ataxia 6	750	755
Undiagnosed ataxia 7	750	720
Undiagnosed ataxia 8	600	636
Undiagnosed ataxia 9	600	575
MSA	800	808
Healthy individual 1	850	765
Healthy individual 2	850	735
